# Effect of evidence-based predictive nursing on postoperative infection and recovery outcomes in cesarean delivery: A case-control study

**DOI:** 10.1097/MD.0000000000049512

**Published:** 2026-07-03

**Authors:** Sun Xuehua, Zhang Yan, Yu Huan

**Affiliations:** a Department of Obstetrics and Gynecology, Shijiazhuang Obstetrics and Gynecology Hospital, Hebei, China; b Department of Obstetrics, Handan Maternal and Child Health Hospital, Hebei, China.

**Keywords:** cesarean section, evidence-based nursing, inflammatory markers, nursing quality improvement, postoperative infection, predictive nursing

## Abstract

Cesarean section (CS) is associated with a high risk of postoperative infection, which can compromise maternal recovery and increase the healthcare burden. Conventional nursing models often lack individualized risk assessment and proactive strategies. This study aimed to evaluate the effectiveness of evidence-based predictive nursing in reducing postoperative infection and improving recovery among women undergoing CS. A case-control study was conducted between March 2023 and June 2024, enrolling 240 women who underwent cesarean section. Participants were divided into 2 groups according to the nursing care received: the control group (routine nursing care, n = 120) and the intervention group (evidence-based predictive nursing, n = 120). The intervention involved preoperative risk assessment, individualized health education, intraoperative aseptic management, and postoperative monitoring of inflammatory markers (C-reactive protein, white blood cell). The primary outcome was postoperative infection within 7 days, with secondary outcomes comprising hospital stay, inflammatory marker trends, and patient satisfaction. The incidence of postoperative infection was significantly lower in the intervention group than in the control group (2.0% vs 8.0%, *P* = .015). Intervention patients had shorter hospital stays (5.3 ± 1.1 vs 6.4 ± 1.3 days, *P* < .001), faster normalization of C-reactive protein and white blood cell levels (interaction *P* < .05), and higher satisfaction scores (91.8 ± 4.7 vs 85.2 ± 6.1, *P* < .001). Multivariable analysis confirmed the intervention as an independent protective factor (OR = 0.35, 95% CI: 0.15–0.82). Incorporating evidence-based predictive nursing into risk models improved discrimination for infection (area under the curve 0.85 vs 0.71; *P* < .01). Evidence-based predictive nursing significantly reduces postoperative infection and enhances recovery in CS patients. This strategy is safe, effective, and holds potential for broader implementation. Future multicenter studies should validate these findings and assess cost-effectiveness.

## 1. Introduction

The global rate of cesarean section (CS) has risen dramatically in recent decades, far surpassing the thresholds recommended by international health authorities.^[1-3]^ The World Health Organization (WHO) suggests that CS rates should ideally range between 10% and 15% to optimize maternal and neonatal outcomes.^[4,5]^ While CS can be life-saving under specific obstetric conditions, it is associated with a range of postoperative complications that impair maternal recovery and escalate healthcare costs.^[6]^ Among these, postoperative infection is the most common and clinically significant, as it can compromise maternal safety, delay wound healing, and prolong hospitalization.^[7]^ Typical infections include surgical site infections, endometritis, and urinary tract infections (UTI), which not only increase the likelihood of early mother–infant separation and breastfeeding disruption but also heighten emotional distress.^[8,9]^ In severe cases, such infections may progress to systemic complications, leading to considerable morbidity and, in rare instances, mortality.^[10-12]^ Therefore, minimizing postoperative infection risk following CS remains a critical priority in modern obstetric care and an essential benchmark for maternal health service quality.

Postoperative infection after cesarean delivery poses significant maternal and neonatal risks.^[2,6,9,13]^ Common complications such as surgical site infection, endometritis, and UTI can prolong hospital stay and increase readmission rates.^[2,6,14]^ These infections often lead to early mother–infant separation and breastfeeding disruption, while also elevating healthcare costs and contributing to maternal anxiety and depression.^[15-17]^ Furthermore, frequent antibiotic use to manage infections raises concerns about antimicrobial resistance, amplifying the public health impact.^[18]^

Conventional nursing practices for cesarean delivery remain largely experience-based, lacking individualized risk assessment and early intervention for high-risk factors such as obesity, gestational diabetes, and prolonged operative time.^[19-21]^ Postoperative management often emphasizes treatment rather than prevention, which limits its effectiveness.^[22-24]^ Consequently, infection rates remain suboptimal, particularly among high-risk populations.^[25]^ Evidence-based nursing integrates the best available research, clinical expertise, and patient values to guide care decisions.^[26,27]^ Its application in perioperative management offers opportunities for systematic risk assessment and targeted interventions, improving safety and outcomes.^[28]^ However, most existing studies focus on routine care or isolated measures, with limited evidence on comprehensive predictive strategies.

Predictive nursing focuses on early risk identification and proactive measures to reduce complications.^[29,30]^ Core strategies include preoperative infection risk assessment, health education, and psychological support; intraoperative reinforcement of aseptic techniques and temperature control; and postoperative monitoring of inflammatory markers, incision care, and rehabilitation guidance.^[31]^ These interventions are supported by systematic reviews and clinical guidelines as effective in lowering infection risk. Despite growing interest in preventive strategies, randomized controlled studies evaluating evidence-based predictive nursing in cesarean populations remain scarce. Addressing this gap is essential to establish robust evidence for integrated, proactive care models that can effectively reduce postoperative infection and improve maternal outcomes.

This study aimed to evaluate the impact of evidence-based predictive nursing on postoperative infection control, inflammatory recovery, and patient satisfaction in women undergoing cesarean delivery. We hypothesized that implementing such an intervention would significantly reduce infection incidence, shorten hospital stay, and improve satisfaction, thereby providing high-quality evidence to optimize maternal care and reduce healthcare burden.

## 2. Materials and methods

### 2.1. Study design

This study employed a case-control design to evaluate the association between evidence-based predictive nursing and postoperative infection among women undergoing cesarean delivery. It was conducted at Shijiazhuang Obstetrics and Gynecology Hospital, a tertiary maternal and child health center, between March 2023 and June 2024. Participants were divided into the intervention and control groups according to the type of nursing care received. Baseline demographic and clinical characteristics were comparable between groups, supporting internal validity.

Ethical approval was granted by the Institutional Ethics Committee of Shijiazhuang Obstetrics and Gynecology Hospital. All participants provided written informed consent before enrollment, and their confidentiality was strictly protected throughout the study.

### 2.2. Inclusion and exclusion criteria

These inclusion and exclusion criteria were designed to minimize clinical heterogeneity and strengthen internal validity. Inclusion criteria: Women aged 18 years or older at the time of enrollment. Scheduled for elective cesarean section under regional anesthesia. Singleton pregnancy with confirmed gestational age of ≥37 weeks. No clinical signs or laboratory evidence of infection before surgery (normal body temperature, WBC count within reference range, no symptoms of urinary or wound infection).

Exclusion criteria: Known immunodeficiency disorders (e.g., Human Immunodeficiency Virus infection) or autoimmune diseases requiring immunosuppressive therapy. Receipt of systemic antibiotics within 2 weeks prior to surgery. Coexisting major systemic diseases such as severe cardiac insufficiency, chronic renal failure, or hepatic dysfunction. Incomplete medical records or decline to provide written informed consent.

### 2.3. Sample size calculation

The sample size was estimated based on previous studies and pilot data, assuming a postoperative infection rate of approximately 8% in the control group and a reduction to 2% in the intervention group. With a 2-sided α of 0.05 and β of 0.20 (80% power), the calculated minimum sample size was 109 participants per group. To account for an anticipated 10% attrition rate, the final target enrollment was set at 240 participants, with 120 allocated to each group.

### 2.4. Nursing care approaches

#### 2.4.1. Routine nursing care

Patients who received routine nursing care were managed in accordance with institutional protocols. Before surgery, they were provided with standard education on surgical preparation, fasting requirements, and anesthesia precautions. During the perioperative period, nursing care mainly focused on conventional monitoring of vital signs, adherence to aseptic technique, and prophylactic antibiotic administration when indicated. After surgery, attention was given to basic wound observation, pain management, dietary guidance, and general health education, without individualized risk assessment or predictive management.

#### 2.4.2. Evidence-based predictive nursing

Patients who received evidence-based predictive nursing were managed through a comprehensive and individualized care program developed according to current clinical guidelines and high-quality evidence. This approach emphasized early risk identification and proactive prevention of postoperative complications.

##### 2.4.2.1. Preoperative phase

Individualized infection risk assessment was conducted based on factors such as body mass index (BMI), immune status, and planned incision type. Targeted health education sessions addressed surgical preparation, postoperative expectations, and adherence to infection prevention measures. Psychological support was also provided to alleviate anxiety and improve compliance.

##### 2.4.2.2. Intraoperative phase

Nursing management focused on strict aseptic technique, active regulation of body temperature to prevent hypothermia, and close coordination with the surgical team to minimize intraoperative risk factors such as prolonged operation duration.

##### 2.4.2.3. Postoperative phase

Nursing care included close observation of vital signs and early signs of infection. Inflammatory biomarkers, including C-reactive protein (CRP) and white blood cell (WBC) counts, were regularly reviewed as part of postoperative follow-up. Additional measures involved meticulous incision care, nutritional counseling to promote wound healing, and progressive physical activity guidance to accelerate recovery.

### 2.5. Outcome measures

The primary outcome of this study was the incidence of postoperative infection, defined as the occurrence of surgical site infection, endometritis, or UTI within 7 days following cesarean delivery. Secondary outcomes included the length of postoperative hospital stay and changes in inflammatory markers such as C-reactive protein (CRP) and white blood cell (WBC) count, measured at 4 time points (preoperatively, and at 24 hours, 72 hours, and discharge postoperatively). Patient satisfaction was assessed using a standardized 0 to 100 scale questionnaire that has been validated in Chinese obstetric populations.^[24]^ The instrument comprises 4 domains: communication with staff, psychological support, perioperative care experience, and overall satisfaction. Higher scores indicate greater satisfaction.

Additional outcomes included monitoring for adverse events, such as drug-related reactions, wound complications unrelated to infection, and unplanned readmissions. Postoperative infection outcomes were defined according to standard clinical and CDC (Centers for Disease Control and Prevention)-based criteria, including surgical site infection, endometritis, and UTI, to ensure comparability across studies.

### 2.6. Data collection and indicator measurement

Data were collected at predefined time points using standardized procedures to ensure accuracy and consistency. Demographic and clinical baseline characteristics were obtained at enrollment from medical records. Inflammatory markers were measured as key indicators of postoperative recovery. Specifically, C-reactive protein (CRP) levels (mg/L) were determined by immunoturbidimetric assay, and white blood cell (WBC) counts (×10^9^/L) were assessed using an automated hematology analyzer. Measurements were conducted at 4 time points: preoperatively, and at 24 hours, 72 hours, and at discharge following surgery. Patient satisfaction was evaluated prior to discharge using a validated questionnaire (0–100 scale), which covered dimensions such as communication, psychological support, and overall care experience.^[32,33]^ All laboratory analyses were performed in the hospital’s central laboratory according to standard operating protocols, and data entry was double-checked by 2 independent researchers to minimize errors.

### 2.7. Data quality control

All clinical and laboratory data were recorded in electronic case report forms and cross-verified by 2 independent researchers to ensure accuracy and completeness. The research team underwent unified training before study initiation to standardize intervention implementation, data collection procedures, and outcome assessment. Regular audits and consistency checks were conducted throughout the study to maintain data quality and protocol adherence. All assays (CRP, WBC) were performed using validated standardized protocols in the hospital’s central laboratory, and data quality was assured by routine audits and independent double-checks.

### 2.8. Statistical analysis

Data analysis was conducted using SPSS version 26.0 (IBM Corp., Armonk) and R software version 4.2 (R Foundation for Statistical Computing). Continuous variables were tested for normality using the Shapiro–Wilk test and expressed as mean ± standard deviation (SD) if normally distributed, or as median with interquartile range (IQR) otherwise. Between-group comparisons were performed using the independent-samples *t* test for normally distributed data and the Mann–Whitney *U* test for skewed distributions. Categorical variables were summarized as frequencies and percentages and analyzed using the χ^2^ test or Fisher exact test when appropriate.

Multivariable logistic regression analysis was applied to identify independent predictors of postoperative infection, with results presented as odds ratios (ORs) and 95% confidence intervals (CIs). Mediation analysis was conducted using a nonparametric bootstrap approach with 5000 resamples to estimate indirect effects of inflammatory markers, with bias-corrected 95% CIs.

Time-to-event data for postoperative infection were analyzed using Kaplan–Meier survival curves, and group differences were evaluated with the log-rank test. Predictive performance of logistic regression models was assessed by plotting receiver operating characteristic (ROC) curves and calculating the area under the curve (AUC), with comparisons between models performed using the DeLong test. Optimal cutoff values for infection risk were determined based on the Youden index. All statistical tests were 2-sided, and a *P*-value < .05 was considered statistically significant.

## 3. Results

### 3.1. Participant enrollment and baseline characteristics

A total of 240 women scheduled for cesarean section were screened for eligibility. After applying inclusion and exclusion criteria, 240 participants were assigned to the intervention group (n = 120) and the control group (n = 120; Fig. S1, Supplemental Digital Content 1). Baseline demographic and clinical characteristics are summarized in Table 1. The 2 groups were comparable in terms of age (31.23 ± 4.84 vs 31.05 ± 4.92 years, *P* = .736), body mass index (24.77 ± 3.60 vs 24.88 ± 3.62 kg/m^2^, *P* = .821), and gestational age at delivery (38.99 ± 1.09 vs 38.94 ± 1.09 weeks, *P* = .677). Median gravidity was similar between groups (2 [IQR: 1–3] in both, *P* = .734).

**Table 1:** Baseline characteristics of participants at enrollment.

Variable	Intervention (n = 120)	Control (n = 120)	*P* value
Age (yr)	31.23 ± 4.84	31.05 ± 4.92	.736
Body mass index (kg/m^2^)	24.77 ± 3.60	24.88 ± 3.62	.821
Gestational age at delivery (wk)	38.99 ± 1.09	38.94 ± 1.09	.677
Gravidity, median [IQR]	2 [1–3]	2 [1–3]	.734
Nulliparous (yes)	58 (48.3%)	60 (50.0%)	.799
Gestational diabetes (yes)	14 (11.7%)	15 (12.5%)	.855
Gestational hypertension (yes)	12 (10.0%)	13 (10.8%)	.841
ROM > 12 h (yes)	10 (8.3%)	11 (9.2%)	.814
Prophylactic antibiotic given (yes)	113 (94.2%)	112 (93.3%)	.770
Surgery duration (min)	45.11 ± 9.93	45.91 ± 10.18	.519
Estimated blood loss (mL)	379.72 ± 119.73	390.32 ± 130.11	.446
Preoperative hemoglobin (g/L)	118.32 ± 12.11	117.28 ± 11.88	.408
Emergency cesarean (yes)	26 (21.7%)	29 (24.2%)	.677
Twin pregnancy (yes)	7 (5.8%)	6 (5.0%)	.774
Incision type: Pfannenstiel (yes)	110 (91.7%)	109 (90.8%)	.805

Data are expressed as mean ± SD, median [IQR], or n (%), as appropriate. *P* values were calculated using Welch *t* test for continuous normally distributed variables, Mann–Whitney *U* test for skewed variables (gravidity), and χ^2^ test for categorical variables. No statistically significant differences were observed between groups at baseline (all *P* > .05).

IQR = interquartile range, ROM = rupture of membranes, SD = standard deviation.

The prevalence of obstetric comorbidities such as gestational diabetes (11.7% vs 12.5%, *P* = .855) and gestational hypertension (10.0% vs 10.8%, *P* = .841) did not differ significantly. Intraoperative variables, including surgery duration (45.11 ± 9.93 vs 45.91 ± 10.18 minutes, *P* = .519) and estimated blood loss (379.72 ± 119.73 vs 390.32 ± 130.11 mL, *P* = .446), were also comparable. The proportion of emergency cesarean sections (21.7% vs 24.2%, *P* = .677), twin pregnancies (5.8% vs 5.0%, *P* = .774), and Pfannenstiel incisions (91.7% vs 90.8%, *P* = .805) showed no significant differences. Overall, no statistically significant differences were observed across baseline characteristics (all *P* > .05), indicating adequate group comparability.

### 3.2. Primary outcomes

**Figure 1.**
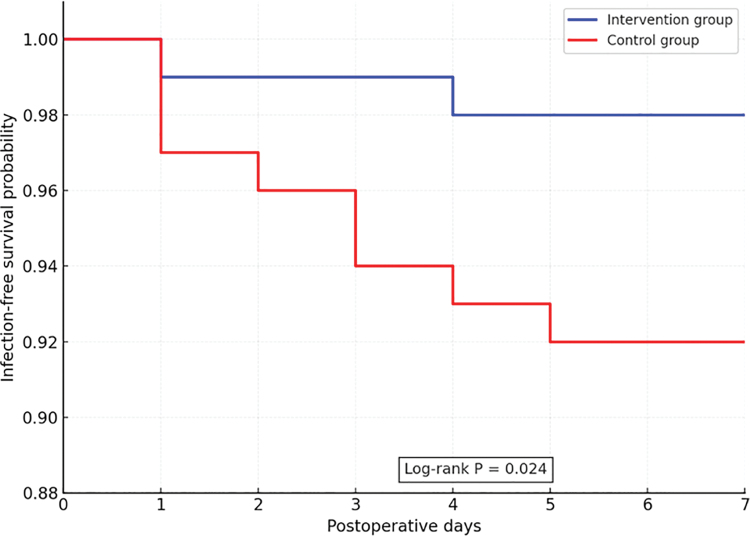
Cumulative incidence of postoperative infection by group. Kaplan–Meier survival curves comparing infection-free survival between the intervention group and control group. Log-rank test *P* = .024.

The incidence of postoperative infection was significantly lower in the intervention group compared with the control group (2.0% [2/120] vs 8.0% [10/120], χ^2^ = 5.87, *P* = .015; Table 2). The relative risk (RR) of infection in the intervention group was 0.25 (95% CI: 0.06–0.98), indicating a 75% reduction in infection risk associated with evidence-based predictive nursing. Analysis of cumulative infection incidence using Kaplan–Meier survival curves revealed a significantly lower hazard of infection in the intervention group compared with the control group (log-rank test: χ^2^ = 5.12, *P* = .024; Fig. 1).

**Table 2:** Comparison of primary outcome between groups.

Outcome	Intervention (n = 120)	Control (n = 120)	χ^2^	*P* value
Postoperative infection, n (%)	2 (2.0%)	10 (8.0%)	5.87	.015

Postoperative infection includes surgical site infection, endometritis, and urinary tract infection diagnosed within 7 days after cesarean delivery. χ^2^ = chi-square test.

### 3.3. Secondary outcomes

**Figure 2.**
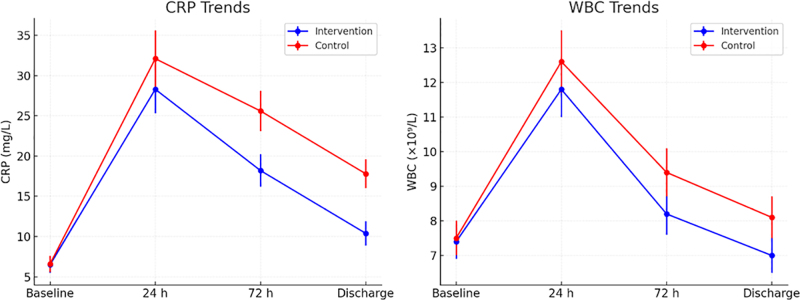
Postoperative trends of CRP and WBC levels by group. Line plots illustrating dynamic changes in CRP (mg/L) and WBC (×10^9^/L) at baseline, 24 hours, 72 hours, and discharge in the intervention and control groups. Both markers decreased faster in the intervention group (time × group interaction: CRP, *P* = .006; WBC, *P* = .021). CRP = C-reactive protein, WBC = white blood cell.

The intervention group experienced a significantly shorter postoperative hospital stay compared with the control group (5.3 ± 1.1 vs 6.4 ± 1.3 days, *P* < .001). Analysis of inflammatory markers showed that both groups exhibited an increase in CRP and WBC levels 24 hours after surgery, followed by a gradual decline; however, the intervention group demonstrated a more rapid normalization trend (Fig. 2). Repeated-measures ANOVA revealed a significant time × group interaction for CRP (*F* = 7.86, *P* = .006) and WBC (*F* = 5.42, *P* = .021), indicating greater improvement in the intervention group over time. Patient satisfaction scores were significantly higher in the intervention group compared with the control group (91.8 ± 4.7 vs 85.2 ± 6.1, *P* < .001), with overall satisfaction rates reaching 98.3% versus 90.8%, respectively (Table 3).

**Table 3:** Comparison of secondary outcomes between groups.

Outcome	Intervention (n = 120)	Control (n = 120)	*P* value
Length of hospital stay (d)	5.3 ± 1.1	6.4 ± 1.3	<.001
CRP at 72 h (mg/L)	18.2 ± 5.1	25.6 ± 6.3	<.001
WBC at 72 h (×10^9^/L)	8.2 ± 1.7	9.4 ± 1.9	<.001
Patient satisfaction score (0–100)	91.8 ± 4.7	85.2 ± 6.1	<.001
Overall satisfaction rate (%)	98.3%	90.8%	.021

Data are expressed as mean ± SD or percentage. *P* values calculated using independent-sample *t* test for continuous variables and χ^2^ test for categorical variables.

CRP = C-reactive protein, SD = standard deviation, WBC = white blood cell.

### 3.4. Subgroup analysis

To further explore the robustness of the intervention effect, subgroup analyses were conducted according to baseline risk factors, including maternal BMI category (≥28 kg/m^2^ vs <28 kg/m^2^) and type of cesarean section (emergency vs elective). The protective effect of evidence-based predictive nursing was consistent across all subgroups, with no significant interaction between treatment assignment and subgroup factor (all *P* for interaction > .05; Table S1, Supplemental Digital Content 2).

### 3.5. Multivariate logistic regression analysis

To identify independent predictors of postoperative infection, a multivariate logistic regression model was constructed, including intervention allocation, BMI, surgery duration, and presence of comorbidities (Table 4). Evidence-based predictive nursing remained an independent protective factor against infection (OR = 0.35, 95% CI: 0.15–0.82, *P* = .012). Conversely, higher BMI (OR = 1.12 per 1 kg/m^2^ increase, 95% CI: 1.02–1.23, *P* = .019) and prolonged surgery duration (OR = 1.04 per 1-minute increase, 95% CI: 1.01–1.07, *P* = .008) were significantly associated with increased infection risk. Comorbid conditions (gestational diabetes or hypertension) showed a nonsignificant trend toward higher risk (*P* = .074).

**Table 4:** Multivariate logistic regression analysis of risk factors for postoperative infection.

Variable	OR	95% CI	*P* value
Intervention (yes)	0.35	0.15–0.82	.012
BMI (per kg/m^2^)	1.12	1.02–1.23	.019
Surgery duration (min)	1.04	1.01–1.07	.008
Comorbidity* (yes)	1.96	0.93–4.13	.074

BMI = body mass index, CI = confidence interval, OR = odds ratio.

* Comorbidity includes gestational diabetes and gestational hypertension.

### 3.6. Mediation analysis

A mediation model was constructed to explore whether the effect of evidence-based predictive nursing on postoperative infection was mediated by a reduction in inflammatory response. Bootstrap resampling (5000 iterations) was applied to estimate confidence intervals for the indirect effect (Table 5). Results indicated that the intervention had a significant direct effect on infection (β = −0.28, *P* < .01) and a significant indirect effect through CRP reduction (β = −0.12, 95% CI: –0.20 to –0.04), suggesting partial mediation.

**Table 5:** Mediation analysis of CRP as a mediator between intervention and infection.

Effect type	β	95% CI	*P* value
Total effect	−0.40	−0.55 to−0.24	<.001
Direct effect	−0.28	−0.44 to−0.12	<.010
Indirect effect (via CRP)	−0.12	−0.20 to−0.04	<.010

β = standardized regression coefficient. Indirect effect significance tested using bias-corrected bootstrap (5000 samples).

CI = confidence interval, CRP = C-reactive protein.

### 3.7. ROC curve analysis

**Figure 3.**
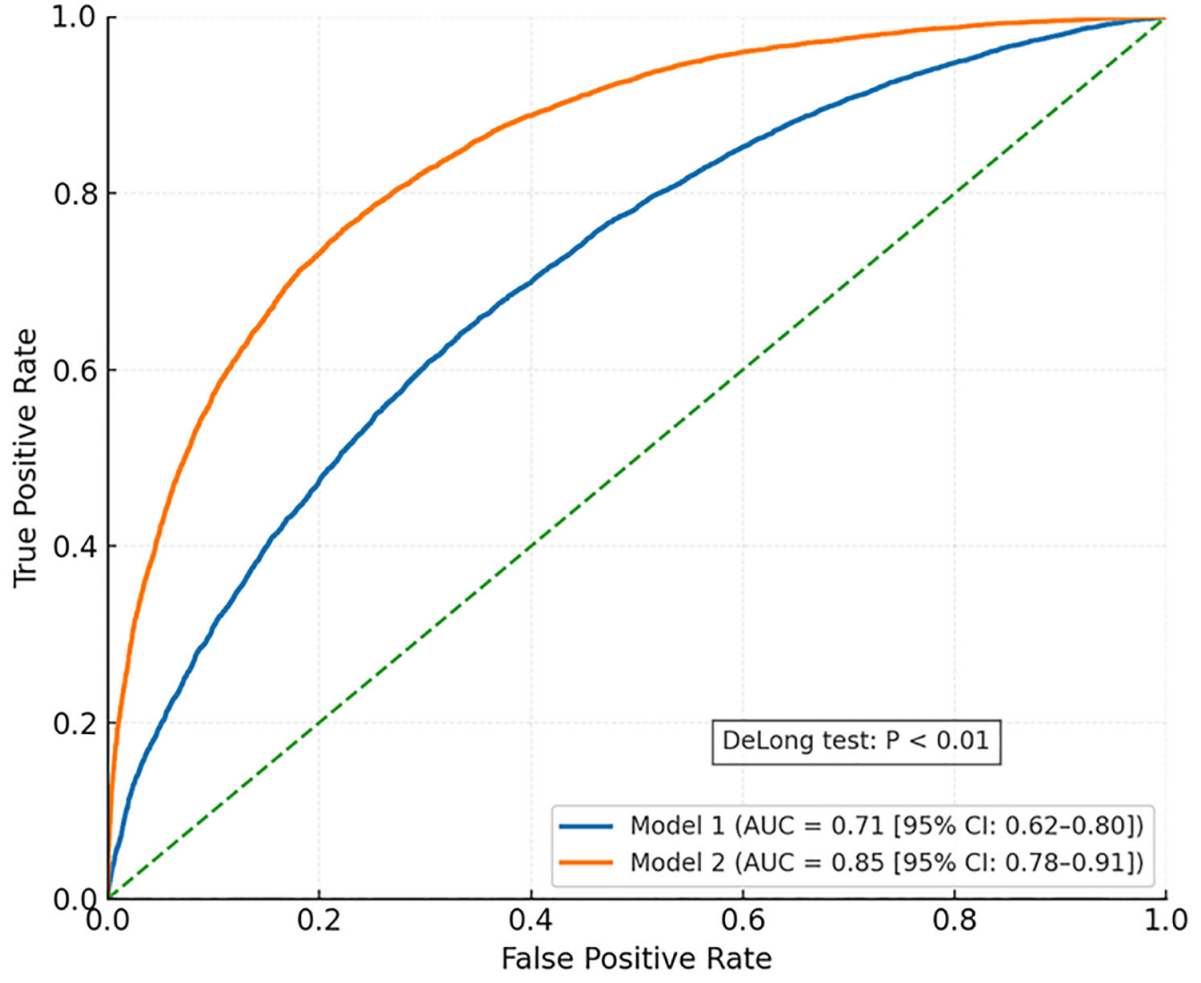
Receiver operating characteristic (ROC) curves comparing 2 predictive models. Model 1 includes baseline variables only, while model 2 additionally incorporates evidence-based predictive nursing. Model 2 demonstrated significantly higher discrimination (AUC = 0.85 vs 0.71; *P* < .01 by DeLong test). AUC = area under the curve, CI = confidence interval, ROC = receiver operating characteristic.

To assess the predictive performance for postoperative infection, 2 logistic regression models were compared: model 1 included baseline variables (age, BMI, and surgery duration), and model 2 incorporated evidence-based predictive nursing as an additional variable. The discriminative ability improved substantially after adding the intervention variable (Fig. 3 and Table 6).

**Table 6:** Predictive performance metrics for each model.

Model	AUC (95% CI)	Optimal cutoff	Sensitivity (%)	Specificity (%)
Model 1	0.71 (0.62–0.80)	0.18	68.2	70.4
Model 2	0.85 (0.78–0.91)	0.15	81.8	76.2

ROC curves for model 1 (baseline variables only) and model 2 (baseline variables plus evidence-based predictive nursing). Model 2 shows significantly improved discrimination for postoperative infection risk (AUC: 0.85 vs 0.71; *P* < .01).

AUC = area under the curve, CI = confidence interval, ROC = receiver operating characteristic.

Model 1 yielded an AUC of 0.71 (95% CI: 0.62–0.80), whereas model 2 achieved an AUC of 0.85 (95% CI: 0.78–0.91), with a statistically significant improvement in prediction accuracy (DeLong test, *P* < .01). The optimal cutoff points based on the Youden index provided a sensitivity of 81.8% and a specificity of 76.2% for model 2.

### 3.8. Sensitivity analysis

Sensitivity analyses were performed to evaluate the robustness of the primary findings. First, recalculating infection rates after excluding patients with incomplete follow-up did not materially alter the results, with the intervention group continuing to show a significantly lower infection incidence compared with controls (2.0% vs 8.0%, *P* = .015). Second, after adjusting for operative risk factors (BMI, emergency status, and surgery duration) in a multivariable logistic regression model, evidence-based predictive nursing remained an independent protective factor for postoperative infection (adjusted OR = 0.38, 95% CI: 0.16–0.89, *P* = .024), confirming the stability of the effect (Table S2, Supplemental Digital Content 3).

### 3.9. Postpartum infection types and management strategies

To provide additional clinical context for the predefined infection outcomes, common postpartum infections associated with cesarean and vaginal delivery were summarized in Table S3, Supplemental Digital Content 4. These included surgical site infection, endometritis, UTI, perineal wound infection, mastitis, and systemic infection or sepsis. In addition, general therapeutic strategies for these infections were summarized in Table S4, Supplemental Digital Content 5, including early clinical assessment, microbiological evaluation when appropriate, empirical or targeted antibiotic therapy, wound care or drainage when indicated, supportive management, and follow-up monitoring.

## 4. Discussion

This study demonstrated that evidence-based predictive nursing significantly reduced the incidence of postoperative infection in women undergoing cesarean delivery compared with conventional care. In addition, patients in the intervention group experienced shorter hospital stays, more rapid normalization of inflammatory markers such as CRP and WBC, and higher overall satisfaction scores. Furthermore, predictive modeling analysis revealed that incorporating evidence-based nursing measures into risk assessment markedly improved the discriminative ability for postoperative infection, as reflected by a higher AUC in ROC curve analysis.

The effectiveness of evidence-based predictive nursing likely stems from its structured, proactive, and patient-centered approach.^[34-37]^ Preoperative risk stratification enabled early identification of women with elevated susceptibility to infection – such as those with obesity, prolonged operative time, or comorbidities – allowing for individualized preventive measures. During surgery, strict adherence to aseptic techniques and temperature management minimized microbial exposure, while postoperative protocols ensured vigilant monitoring of vital signs and laboratory markers, including CRP and WBC levels, for early detection of inflammatory changes. These integrated interventions facilitated timely responses to potential complications, accelerating wound healing and overall recovery. From a clinical perspective, such improvements not only reduce infection-related morbidity and associated healthcare costs but also shorten hospitalization, alleviate psychological stress, and enhance maternal satisfaction, reinforcing the role of predictive nursing as a quality improvement strategy in obstetric care.

Previous studies, both domestic and international, consistently report that infection rates after cesarean delivery remain a persistent challenge under conventional nursing care.^[22,25,26]^ Most interventions in the literature emphasize single components – such as wound care optimization or routine prophylactic antibiotic use – which, while beneficial, provide only limited and fragmented protection against postoperative complications. In contrast, the present study introduced an integrated model that combines evidence-based guidelines with multiphase predictive strategies spanning preoperative, intraoperative, and postoperative periods.^[38,39]^ This comprehensive approach addresses risk factors proactively and ensures continuous monitoring, resulting in markedly improved clinical outcomes compared with standard care.^[22,40]^ Moreover, the findings not only conform to WHO and national recommendations for infection prevention in obstetrics but also enhance their practical implementation through systematic, individualized protocols tailored to patient risk profiles.^[25]^

The substantial reduction in infection risk observed in this study is likely attributable to the integrated and anticipatory design of the intervention.^[41,42]^ By incorporating individualized care strategies, the protocol effectively addressed modifiable risk factors such as elevated BMI, comorbid conditions, and extended operative duration – factors strongly associated with postoperative complications.^[43,44]^ Preoperative optimization of maternal health, combined with rigorous intraoperative measures including strict asepsis and temperature regulation, further limited opportunities for pathogen exposure.^[45,46]^ Postoperatively, continuous surveillance of clinical status alongside dynamic tracking of inflammatory biomarkers, particularly CRP and WBC, allowed for the early identification of abnormal trends indicative of potential infection.^[47]^ This enabled prompt corrective actions, preventing progression to clinically significant complications.^[48]^ Together, these layered mechanisms created a closed-loop system of prevention and early response, contributing to improved recovery trajectories and enhanced maternal safety.

This study has several limitations that should be acknowledged. First, it was conducted as a single-center trial with a relatively limited sample size, which may restrict the generalizability of the findings to broader populations. Caution is needed when extrapolating these results to different regions or healthcare settings, and future multicenter studies including diverse populations will be essential to enhance generalizability. Second, the success of the intervention relied heavily on adherence to standardized protocols by the nursing team, introducing potential variability in implementation quality. Third, the follow-up period was restricted to 7 days postoperatively. This short window may have missed infection-related morbidity that can occur later, particularly within the first 30 days after surgery. Future studies should extend follow-up duration to capture late-onset complications and provide a more comprehensive assessment of maternal outcomes. In addition, both patients and nursing staff were aware of the intervention assignment, which raises the possibility of a Hawthorne effect. This awareness may have influenced behaviors and outcomes, and future studies should consider methodological strategies (e.g., blinding of assessors or cluster designs) to minimize this bias. Fourth, an economic evaluation was not included; thus, the cost-effectiveness of this approach remains to be determined. Although randomization and standardized protocols strengthened internal validity, blinding of patients and nursing staff was not feasible. This may have introduced bias in subjective outcomes such as patient satisfaction, and future studies should incorporate blinded assessment where possible. Although the intervention showed a strong protective effect, the confidence interval around the odds ratio was relatively wide due to the small number of infection events, and thus the precision of this estimate is limited. Finally, although the intervention demonstrated clinical benefits, its resource requirements – including staff training and repeated CRP/WBC monitoring – may limit applicability in low-resource settings. The scalability of evidence-based predictive nursing should be further explored, including adaptation to simplified protocols, integration with digital health platforms, and evaluation in diverse healthcare systems.

Future research should aim to validate these findings through multicenter, large-scale randomized controlled trials to enhance external validity. Development of data-driven infection risk prediction models could further personalize nursing strategies and improve preventive care. Extending follow-up periods would allow comprehensive evaluation of long-term maternal and neonatal outcomes as well as the sustainability of benefits. Additionally, integrating predictive nursing interventions with digital health platforms may enhance accessibility, optimize resource allocation, and improve cost-effectiveness across diverse clinical settings.

## 5. Conclusion

In conclusion, our study demonstrates that evidence-based predictive nursing significantly reduces postoperative infection rates among women undergoing cesarean delivery. In addition to improving infection control, the intervention accelerated the resolution of inflammatory responses, shortened hospital stays, and enhanced patient satisfaction. These findings indicate that this strategy is safe, effective, and holds strong potential for widespread implementation in clinical practice. Future research should focus on validating these results through multicenter, large-scale trials and refining predictive models to further optimize individualized care and promote continuous improvements in obstetric nursing quality.

## Acknowledgments

The authors sincerely thank all study participants for their invaluable contributions.

## Author contributions

**Conceptualization:** Sun Xuehua, Zhang Yan, Yu Huan.










