# Supplementary figures and images for: Effect of evidence-based predictive nursing on postoperative infection and recovery outcomes in cesarean delivery: A case-control study
Source: Medicine (Baltimore). 2026 Jul 3;105(27):e49512. doi: 10.1097/MD.0000000000049512 (PMC13337019; doi:10.1097/MD.0000000000049512)

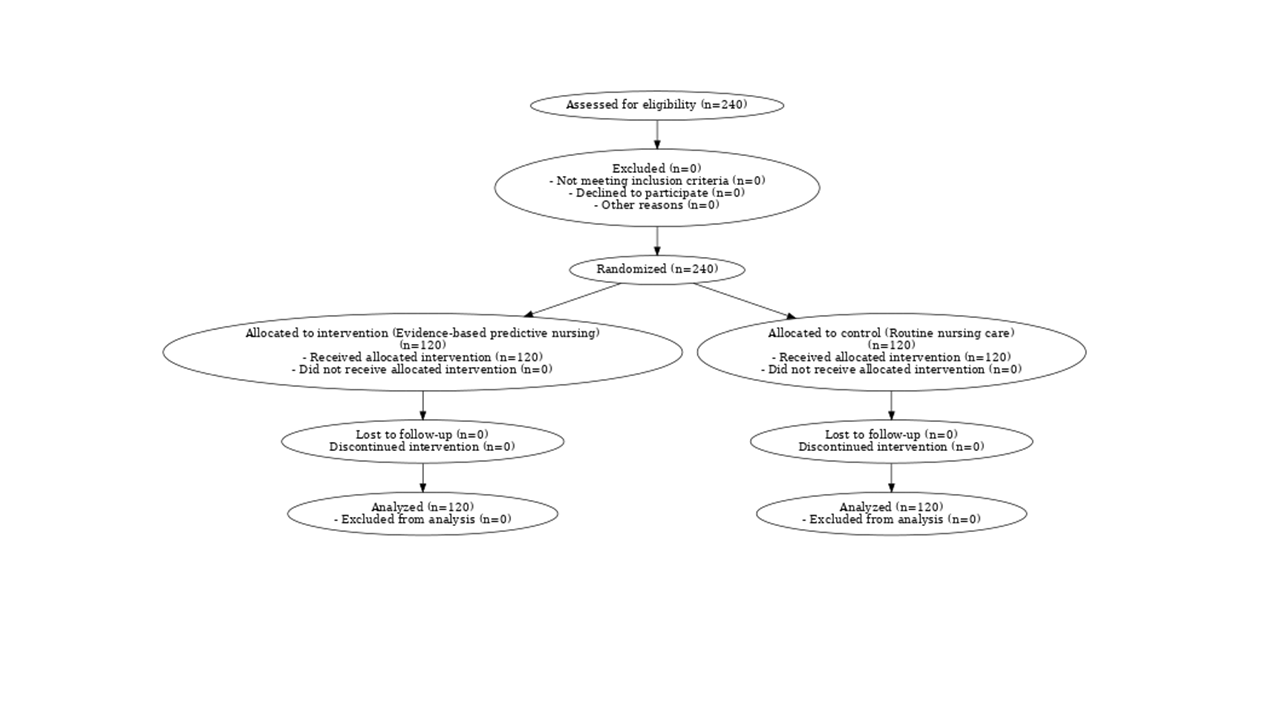

Supplement: Supplementary file 1 [file medi-105-e49512-s001.tif]
