# Supplementary material for: Effect of evidence-based predictive nursing on postoperative infection and recovery outcomes in cesarean delivery: A case-control study
Source: Medicine (Baltimore). 2026 Jul 3;105(27):e49512. doi: 10.1097/MD.0000000000049512 (PMC13337019; doi:10.1097/MD.0000000000049512)
Supplement: Supplementary file 2 [file medi-105-e49512-s002.docx]

**Supplementary Table S1. Subgroup analysis of intervention effect on postoperative infection**

| **Subgroup** | **Intervention Infection (%)** | **Control Infection (%)** | **Relative Risk (95% CI)** | **P value** | **P for interaction** |
| --- | --- | --- | --- | --- | --- |
| **BMI category** |  |  |  |  |  |
| <28 kg/m² | 1/92 (1.1%) | 6/94 (6.4%) | 0.17 (0.02–1.36) | 0.065 |  |
| ≥28 kg/m² | 1/28 (3.6%) | 4/26 (15.4%) | 0.23 (0.03–1.64) | 0.091 | 0.421 |
| **Cesarean type** |  |  |  |  |  |
| Elective | 1/94 (1.1%) | 5/91 (5.5%) | 0.20 (0.02–1.64) | 0.083 |  |
| Emergency | 1/26 (3.8%) | 5/29 (17.2%) | 0.22 (0.03–1.52) | 0.095 | 0.438 |

**Table Note:** Data are n (%) unless otherwise indicated. Relative risk and 95% CI estimated using log-binomial regression within each subgroup. P for interaction was derived from multiplicative interaction terms in the full model.
