# Supplementary material for: Effect of evidence-based predictive nursing on postoperative infection and recovery outcomes in cesarean delivery: A case-control study
Source: Medicine (Baltimore). 2026 Jul 3;105(27):e49512. doi: 10.1097/MD.0000000000049512 (PMC13337019; doi:10.1097/MD.0000000000049512)
Supplement: Supplementary file 3 [file medi-105-e49512-s003.docx]

**Supplementary Table S2. Sensitivity analysis of intervention effect on postoperative infection**

| **Analysis scenario** | **Intervention infection (%)** | **Control infection (%)** | **Adjusted OR (95% CI)** | **P value** |
| --- | --- | --- | --- | --- |
| Primary analysis | 2.0% | 8.0% | 0.35 (0.15–0.82) | 0.012 |
| Excluding incomplete follow-up | 2.0% | 8.0% | 0.35 (0.15–0.82) | 0.015 |
| Adjusted for operative risk factors* | — | — | 0.38 (0.16–0.89) | 0.024 |

*Risk factors include BMI, emergency cesarean section, and surgery duration.
