# Supplementary material for: Effect of evidence-based predictive nursing on postoperative infection and recovery outcomes in cesarean delivery: A case-control study
Source: Medicine (Baltimore). 2026 Jul 3;105(27):e49512. doi: 10.1097/MD.0000000000049512 (PMC13337019; doi:10.1097/MD.0000000000049512)
Supplement: Supplementary file 4 [file medi-105-e49512-s004.docx]

**Supplementary Table S3. Common postpartum infections after cesarean delivery and vaginal delivery**

| **Infection type** | **Cesarean delivery** | **Vaginal delivery** | **Clinical relevance** |
| --- | --- | --- | --- |
| Surgical site infection | Commonly associated with abdominal and uterine incisions; may present as wound erythema, swelling, pain, discharge, or dehiscence | Not applicable to abdominal incision; however, perineal wound infection may occur after episiotomy or perineal laceration | Important cause of delayed wound healing, prolonged hospital stay, and readmission |
| Endometritis | More frequent after cesarean delivery because of intrauterine manipulation, surgical exposure, and tissue trauma | May occur after prolonged labor, premature rupture of membranes, retained products of conception, or repeated vaginal examinations | May present with fever, uterine tenderness, foul-smelling lochia, and elevated inflammatory markers |
| Urinary tract infection | May be related to perioperative catheterization, reduced mobility, and postoperative urinary retention | May occur because of catheterization, perineal trauma, urinary retention, or postpartum voiding dysfunction | Can delay recovery and may progress to pyelonephritis if untreated |
| Perineal wound infection | Generally not applicable unless concomitant perineal trauma exists | May occur after episiotomy or perineal laceration | Can cause pain, delayed healing, and impaired mobility |
| Mastitis | May occur if breastfeeding is delayed or disrupted by postoperative pain, maternal fatigue, or mother–infant separation | May also occur during breastfeeding, especially with milk stasis or nipple trauma | May affect breastfeeding continuation and maternal comfort |
| Systemic infection or sepsis | Rare but serious; may develop from uncontrolled surgical site infection, endometritis, or urinary tract infection | Rare but possible, especially when genital tract or urinary infection is not recognized early | Requires urgent recognition and treatment to prevent severe maternal morbidity |

**Table note:** This table summarizes common postpartum infections for clinical context. The primary outcome of the present study focused on postoperative infection within 7 days after cesarean delivery, including surgical site infection, endometritis, and urinary tract infection.
