# Supplementary material for: Effect of evidence-based predictive nursing on postoperative infection and recovery outcomes in cesarean delivery: A case-control study
Source: Medicine (Baltimore). 2026 Jul 3;105(27):e49512. doi: 10.1097/MD.0000000000049512 (PMC13337019; doi:10.1097/MD.0000000000049512)
Supplement: Supplementary file 5 [file medi-105-e49512-s005.docx]

**Supplementary Table S4. General therapeutic strategies for common postpartum infections**

| **Infection type** | **Main therapeutic strategy** | **Additional management considerations** |
| --- | --- | --- |
| Surgical site infection | Early wound assessment, empirical antibiotics when bacterial infection is suspected, and targeted antibiotics based on culture results when available | Wound drainage, debridement, dressing changes, glycemic control, pain management, and close follow-up when indicated |
| Endometritis | Prompt broad-spectrum antibiotic therapy according to institutional protocols and local antimicrobial resistance patterns | Assessment for retained products of conception, monitoring of fever and inflammatory markers, and escalation of care if clinical response is inadequate |
| Urinary tract infection | Urinalysis and urine culture when appropriate, followed by empirical or targeted antibiotic therapy | Encourage hydration, assess urinary retention, remove unnecessary catheterization, and monitor for pyelonephritis |
| Perineal wound infection | Local wound care, pain control, and antibiotics when cellulitis, purulent discharge, or systemic symptoms are present | Evaluate wound separation, abscess formation, and the need for drainage or secondary repair |
| Mastitis | Continued breastfeeding or milk expression, breast emptying, analgesia, and antibiotics when bacterial mastitis is suspected | Evaluate for breast abscess if symptoms persist or a localized mass develops |
| Systemic infection or sepsis | Immediate clinical assessment, blood cultures when indicated, broad-spectrum intravenous antibiotics, fluid resuscitation, and source control | Requires multidisciplinary management and close monitoring of vital signs, organ function, and laboratory parameters |

**Table note:** Therapeutic strategies should be individualized according to clinical severity, microbiological findings, local antimicrobial policies, breastfeeding status, and institutional guidelines. This table is intended to provide general clinical context and does not replace local treatment protocols.
